# Reduced penetrance of the *PSEN1* H163Y autosomal dominant Alzheimer mutation: a 22-year follow-up study

**DOI:** 10.1186/s13195-018-0374-y

**Published:** 2018-05-10

**Authors:** Steinunn Thordardottir, Elena Rodriguez-Vieitez, Ove Almkvist, Daniel Ferreira, Laure Saint-Aubert, Anne Kinhult-Ståhlbom, Håkan Thonberg, Michael Schöll, Eric Westman, Anders Wall, Maria Eriksdotter, Henrik Zetterberg, Kaj Blennow, Agneta Nordberg, Caroline Graff

**Affiliations:** 1 Karolinska Institutet, Department of Neurobiology, Care Sciences and Society, Center for Alzheimer Research, Division of Neurogeriatrics, 141 57 Huddinge, Sweden; 2 Theme Aging, Karolinska University Hospital Huddinge, 141 86 Stockholm, Sweden; 3 Karolinska Institutet, Department of Neurobiology, Care Sciences and Society, Center for Alzheimer Research, Division of Translational Alzheimer Neurobiology, 141 57 Huddinge, Sweden; 4 Department of Psychology, Stockholm University, 106 91 Stockholm, Sweden; 5 Karolinska Institutet, Department of Neurobiology, Care Sciences and Society, Center for Alzheimer Research, Division of Clinical Geriatrics, 141 57 Huddinge, Sweden; 6 Toulouse NeuroImaging Center, Université de Toulouse, Inserm, UPS, Toulouse, France; 7 Wallenberg Centre for Molecular and Translational Medicine and the Department of Psychiatry and Neurochemistry, University of Gothenburg, 413 45 Gothenburg, Sweden; 8 Clinical Memory Research Unit, Lund University, 212 24 Malmö, Sweden; 9 Uppsala University, Department of Surgical Sciences, Section of Nuclear Medicine & PET, 751 85 Uppsala, Sweden; 10 Institute of Neuroscience and Physiology, Department of Psychiatry and Neurochemistry, The Sahlgrenska Academy at the University of Gothenburg, 431 80 Mölndal, Sweden; 11 UCL Institute of Neurology, Queen Square, London, WC1N 3BG UK; 12 UK Dementia Research Institute at UCL, London, WC1N 3BG UK; 13 Clinical Neurochemistry Laboratory, Sahlgrenska University Hospital, Mölndal, 431 80 Mölndal, Sweden

**Keywords:** Autosomal dominant Alzheimer’s disease, CSF, [^18^F]fluorodeoxyglucose PET, [^11^C]Pittsburgh compound B PET, Reduced penetrance

## Abstract

**Background:**

The range of onset ages within some *PSEN1* families is wide, and a few cases of reduced penetrance of *PSEN1* mutations have been reported. However, published data on reduced penetrance have been limited to clinical histories, often collected retrospectively and lacking biomarker information. We present a case of reduced penetrance of the *PSEN1* H163Y mutation in a carrier prospectively followed for 22 years.

**Methods:**

Two brothers (A and B), both carriers of the H163Y mutation, were followed between 1995 and 2017. They underwent repeated clinical evaluations, neuropsychological assessments, and cerebrospinal fluid analyses, as well as brain imaging examinations with structural magnetic resonance, [^18^F]fluorodeoxyglucose positron emission tomography, and [^11^C]Pittsburgh compound B positron emission tomography.

**Results:**

Brother A was followed between 44 and 64 years of age. Cognitive symptoms due to Alzheimer’s disease set in at the age of 54. Gradual worsening of symptoms resulted in admittance to a nursing home owing to dependence on others for all activities of daily living. He showed a curvilinear decline in cognitive function on neuropsychological tests, and changes on magnetic resonance imaging, positron emission tomography, and biomarkers in the cerebrospinal fluid supported a clinical diagnosis of Alzheimer’s disease. Brother A died at the age of 64 and fulfilled the criteria for definitive Alzheimer’s disease according to neuropathological examination results. Brother B was followed between the ages of 43 and 65 and showed no cognitive deterioration on repeated neuropsychological test occasions. In addition, no biomarker evidence of Alzheimer’s disease pathology was detected, either on imaging examinations or in cerebrospinal fluid.

**Conclusions:**

The average (SD) age of symptom onset for *PSEN1* H163Y is 51 ± 7 years according to previous studies. However, we present a case of a biomarker-verified reduction in penetrance in a mutation carrier who was still symptom-free at the age of 65. This suggests that other genetic, epigenetic, and/or environmental factors modify the onset age.

**Electronic supplementary material:**

The online version of this article (10.1186/s13195-018-0374-y) contains supplementary material, which is available to authorized users.

## Background

Familial Alzheimer’s disease (FAD) is an early-onset form of Alzheimer’s disease inherited in an autosomal dominant fashion. Currently, mutations causing FAD have been found in three genes: the amyloid precursor protein (*APP*) gene on chromosome 21 [1–3], the presenilin 1 (*PSEN1*) gene on chromosome 14 [4], and the presenilin 2 (*PSEN2*) gene on chromosome 1 [5]. These FAD mutations are generally considered to be 100% penetrant and have a relatively predictable age of clinical symptom onset [6, 7]. There are significant differences between FAD mutation types in the expected age of clinical symptom onset [8, 9] as well as in clinical presentation and cognitive phenotype [10–12]. There is also variability in age at clinical onset within a given family [9], suggesting that other genetic, epigenetic, environmental, or stochastic factors may influence disease onset [9]; however, these additional modifying factors are not well known. The discovery of FAD mutations has provided new insights into the pathogenesis of Alzheimer’s disease, rendering support for the amyloid cascade hypothesis. According to this hypothesis, it is the accumulation of amyloid-β peptide in the brain that primarily initiates and drives the disease process [13].

Mutations in *APP*, *PSEN1*, and *PSEN2* all have an effect on the production, clearance, or conformational structure of the amyloid-β peptide [14, 15]. Mutations in *PSEN1* are the most common known mutations causing FAD [16, 17]. The *PSEN1* gene codes for the presenilin 1 protein, a subunit of a large enzymatic complex, the γ-secretase, that plays a role in amyloid-β production. The amyloid-β peptide is released from the amyloid precursor protein after this protein is cleaved by the β-secretase and subsequently by the γ-secretase enzymes. Presenilin 1 is the proteolytic subunit of γ-secretase and is responsible for the cleavage of amyloid precursor protein, releasing amyloid-β peptides of various lengths [18]. Most commonly, γ-secretase cleavage results in amyloid-β peptides that are 40 amino acids in length (amyloid-β_40_). A less abundant and more fibrillogenic form of the amyloid-β peptide is 42 amino acids long (amyloid-β_42_). *PSEN1* mutations cause a relative increase in the ratio of amyloid-β_42_ to amyloid-β_40_ through an increase in amyloid-β_42_ production, a decrease in amyloid-β_40_ production, or both [15]. The mechanisms behind the changes in the balance between different lengths of amyloid-β peptides observed in *PSEN1* mutations have been further elucidated in recent studies. These mutations cause a variable inhibitory effect at the initial endoproteolytic ε-cleavage step by the γ-secretase and a consistent effect on the consequent γ-secretase cleavage steps, causing a premature release of longer amyloid-β peptides. Finally, they affect the initial position of the ε-site (i.e., whether the γ-secretase preferentially starts its cleavage at positions 49–50 or 51–50 in the *APP* sequence) [18]. These findings have been further corroborated in a study on γ-secretase activity in brain samples from carriers of *PSEN1* mutations showing no loss of overall γ-secretase activity in carriers, but rather a dysfunction of the γ-secretase, leading to production of longer amyloid-β peptides [19].

To date, over 240 mutations in the *PSEN1* gene have been reported [20], most of which are pathogenic and lead to FAD. Reduced penetrance of *PSEN1* mutations is rare, but there are a few cases described in the literature. The median age at onset for the I143F mutation is 55 years, but a carrier of this mutation was symptom-free at the age of 68 [21]. In another report, the A79V mutation (with a mean onset age of 64 years) was not yet penetrant in a 76-year-old mutation carrier [22]. In both of these cases, the symptom-free mutation carriers had the *APOE* ε3/ε3 genotype. In a family carrying the M139V mutation, there were 34 years between the individual with the youngest onset (35 years old) and the individual with the oldest onset (69 years old), and they both had the same *APOE* genotype (ε3/ε4) [23]. On immunostaining of postmortem frozen brain tissue sections, the individual with the earliest onset in the M139V family had about a twofold higher amount of amyloid-β deposits, neurofibrillary tangles, and neuronal loss than the individual with later onset. Finally, three cases of FAD due to the K239N mutation have been described, with a range of symptom onset from 42 to 71 years (based on clinical history). All of the three K239N mutation carriers had the *APOE* ε3/ε3 genotype [24]. These variations in onset and rare cases of reduced penetrance of *PSEN1* mutations are intriguing and suggest the presence of factors, genetic or environmental, that have a modifying effect on the disease process.

In this report, we present a case of reduced penetrance of the *PSEN1* H163Y mutation. The *PSEN1* H163Y mutation was first described by Clark et al*.* in 1995 and to date has been found in only a single Swedish family [25]. In a culture of cells derived from monkey kidney tissue (COS-1 cells) transfected with complementary DNA (cDNA) encoding human *PSEN1* H163Y, the mutation caused a 2.1-fold increase in the ratio of amyloid-β_42_ to total amyloid-β compared with wild-type *PSEN1* [26]. The mean age at symptom onset for the H163Y mutation is 51 years, with an SD of 7 years, based on 11 affected individuals (unpublished data, Thordardottir S). A case of reduced penetrance of this mutation was previously reported in a family member who was an obligate mutation carrier, because an offspring was confirmed to carry the mutation by DNA analysis and later died of the disease. This obligate carrier died at the age of 67 without any cognitive symptoms being reported by relatives [27].

In this paper, we report prospectively collected follow-up data on two *PSEN1* H163Y mutation carrier brothers over a period of 22 years, including repeated clinical examinations and collection of biomarkers. The brothers were born 1 year apart, and during the study, one brother developed Alzheimer’s disease and died in a nursing home, whereas the other brother remained symptom-free and without pathological biomarkers. To our knowledge, this is the first case of reduced penetrance of a *PSEN1* mutation described in the literature where the subjects have been followed prospectively with thorough investigations over an extended period of time.

## Methods

### Study design and participants

Two brothers, A and B, both carriers of the *PSEN1* H163Y mutation, were part of a larger longitudinal clinical and experimental study on FAD that has been ongoing at Karolinska Institutet since 1993. All participants in this longitudinal study were recruited through the Genetics Unit, which provides genetic counseling in the Memory Clinic at Karolinska University Hospital in Huddinge, Sweden. The FAD study is a prospective study where subjects undergo repeated examinations over time, including clinical evaluation, a comprehensive neuropsychological assessment, neuroimaging, electroencephalography, and collection of blood and cerebrospinal fluid (CSF). The criteria of the International Working Group on Mild Cognitive Impairment [28] were used for the diagnosis of mild cognitive impairment (MCI), and the criteria for dementia due to Alzheimer’s disease according to the National Institute on Aging-Alzheimer’s Association workgroups were used for Alzheimer’s disease diagnosis [29]. Some of the MRI, CSF, and positron emission tomography (PET) data from the two brothers included in this study has been presented separately in previous publications from the FAD study, and brother B was mentioned to be an outlier in those papers [30–33]. Brothers A and B presented in this report entered the study in 1995 when they were 44 and 43 years old, respectively. Brother A was examined on 10 separate occasions (from 1995 to 2015), and brother B was examined on 12 separate occasions (from 1995 to 2017) (*see* Table 1).

**Table 1:** Overview of participation of brothers A and B in familial Alzheimer’s disease study

	Year	Brother A	Brother B
Baseline	1995	NPA, MRI, CSF	NPA, CSF
+ 1 year	1996	FDG-PET	FDG-PET
+ 2 years	1997	NPA, MRI	NPA
+ 3 years	1998	FDG-PET	FDG-PET
+ 6 years	2001	NPA, MRI	NPA
+ 11 years	2006	NPA, MRI, CSF, FDG-PET	NPA, CSF, FDG-PET
+ 12 years	2007	Interview	
+ 13 years	2008	NPA, FDG-PET, PiB-PET	
+ 14 years	2009	NPA, MRI, FDG-PET, PiB-PET	NPA, MRI, FDG-PET, PiB-PET
+ 16 years	2011		NPA
+ 17 years	2012		PiB-PET
+ 18 years	2013		NPA
+ 20 years	2015	Interview, MMSE	Interview, MMSE
+ 22 years	2017		NPA

*Abbreviations: CSF* Cerebrospinal fluid sampling, *FDG-PET* [^18^F]fluorodeoxyglucose PET, *MMSE* Mini Mental State Examination, *MRI* Magnetic resonance imaging, *NPA* Neuropsychological assessment, *PiB-PET* [^11^C]Pittsburgh compound B positron emission tomography, MRI Magnetic resonance imaging

The rows in the table indicate the year in which different examinations were performed. Brother A was diagnosed with mild cognitive impairment in 2006 and with dementia due to Alzheimer’s disease in 2007 (based on clinical symptoms)

Both subjects received genetic counseling in conjunction with their participation in the study. Initially, the brothers were blind to their mutation status, and the same applied to the clinicians and researchers involved in the study. Brother A opted for diagnostic genetic testing in 2008 after he was diagnosed with Alzheimer’s disease. Brother B requested a presymptomatic genetic test in 2009. *APOE* genotyping was also performed, revealing that brother A had the ε2/ε4 genotype and brother B had the ε2/ε3 genotype.

### Genetic analysis

#### Apolipoprotein E

The *APOE* genotyping was performed for single-nucleotide polymorphisms (SNPs) rs7412 and rs429358 using TaqMan® SNP genotyping assays (Applied Biosystems, Foster City, CA, USA) according to the manufacturer’s protocol. The amplified products were run on the 7500 fast Real-Time PCR System (Applied Biosystems, Foster City, CA, USA).

#### Mutation analyses in *PSEN1*

To confirm the H163Y mutation in *PSEN1*, exon 6 was sequenced [25]. DNA was amplified using AmpliTaq Gold® 360 PCR Master Mix (Applied Biosystems). Primer sequences and PCR conditions are available upon request. The BigDye® Terminator v3.1 Cycle Sequencing Kit (Applied Biosystems) was used for Sanger sequencing. Exon 6 in *PSEN1* was sequenced in both directions and analyzed on an ABI 3500 Genetic Analyzer (Applied Biosystems).

For both brothers A and B, multiple DNA samples extracted on separate occasions from both blood (two different sample years) and skin biopsies (two different sample dates) were sequenced using three different methods: Sanger sequencing, next-generation gene panel sequencing, and whole-genome sequencing. Finally, cDNA extracted from fibroblast cultures was sequenced and confirmed the presence of both the wild-type transcript and the H163Y mutation transcript.

### Neuropsychological assessment

The same set of 12 neuropsychological tests was used for brothers A and B in all assessments and administered by the same psychologist. The Information and Similarities tests [34] were used to assess verbal ability; the Block Design [34, 35] and Rey-Osterrieth copy [36] tests were used to assess visuospatial ability; the Digit Span forward [34, 35] and Corsi Span [36] tests were used to assess immediate memory; the Rey Auditory Verbal Learning (RAVL) total learning and 30-minute retention as well as Rey-Osterrieth 30-minute retention [36] tests were used to assess episodic memory; the Trail Making Test A [36] was used to assess attention; and the Digit Symbol [34, 35] and Trail Making Test B [36] were used to assess executive function. A measure of current global cognitive function was calculated using five tests: Information, Similarities, Block Design, Digit Span, and Digit Symbol [34, 35]. Premorbid global cognitive function was estimated using the Swedish New Adult Reading Test [37]. All raw scores were converted to z-scores using a reference group of healthy adults from the Karolinska University Hospital in Huddinge, Sweden [38].

### CSF sampling and analysis

The CSF samples were collected by lumbar puncture in the L3-L4 or L4-L5 interspace. Both brothers underwent a lumbar puncture on two occasions, first in 1995 at baseline, when the brothers were 44 (A) and 43 years old (B), and again in 2006, 11 years after baseline, when they were 55 (A) and 54 years old (B). Immediately after collection, the CSF was centrifuged at 3000 × *g* at + 4 °C for 10 minutes. The supernatant was pipetted off, aliquoted into polypropylene cryotubes, and stored at − 80 °C. The aliquots had been thawed and refrozen once before being thawed for analysis in this study. The CSF samples were all analyzed at the same time in 2012 at the Clinical Neurochemistry Laboratory at the Sahlgrenska University Hospital, Mölndal, Sweden, by board-certified laboratory technicians blind to clinical data. All analytical procedures were performed according to protocols accredited by the Swedish Board for Accreditation and Conformity Assessment. CSF amyloid-β_42_ was analyzed by using electrochemiluminescence technology with the MS6000 Human Aβ 3-Plex Ultra-Sensitive Kit (Meso Scale Discovery, Gaithersburg, MD, USA) [39]. CSF total tau (T-tau) protein was determined using a sandwich enzyme-linked immunosorbent assay (ELISA) (INNOTEST hTAU-Ag; Fujirebio Europe, Ghent, Belgium) specifically constructed to measure all tau isoforms regardless of phosphorylation status, as previously described [40], whereas P-tau (tau phosphorylated at threonine 181) was measured using the INNOTEST® PHOSPHO-TAU_(181P)_ ELISA (Fujirebio Europe), as described previously in detail [41]. Owing to unusual results obtained from the CSF amyloid-β_42_ analysis in 2012, the samples were reanalyzed for CSF amyloid-β_42_ levels in 2017 using the same method.

### PET image acquisition

The brothers underwent both [^18^F]fluorodeoxyglucose (FDG) and [^11^C]Pittsburgh compound B (PiB) PET at several time points (Table 1); some of these longitudinal PET data have been published previously [33]. All PET examinations were performed at the Uppsala PET Centre, University of Uppsala, Sweden. The first two FDG scans were acquired on a GEMS 2048-15B scanner (GE Medical Systems, Milwaukee, WI, USA) for brother A and a GEMS 4096-15WB scanner for brother B. All other acquisitions (FDG and PiB scans) were performed on an ECAT EXACT HR+ (Siemens CTI; Erlangen, Germany) scanner or a Discovery ST PET/CT (GE Medical Systems) scanner [33]. The mean injected doses were approximately 3 MBq/kg for FDG and 4 MBq/kg for PiB. Sum images were created for both FDG (30–45 minutes) and PiB (40–60 minutes) scans and were used for subsequent image analyses. To interpret the regional values of FDG and PiB PET uptake, corresponding z-score values were calculated with respect to a group of 14 cognitively normal noncarriers who had previously undergone both FDG and PiB PET scanning [30] at the Uppsala PET Centre, with images acquired on an ECAT EXACT HR+ or a Discovery ST PET/CT scanner. The group of noncarriers had a median age of 57 years (range 35–71); 4 of the 14 were female, and all had Mini Mental State Examination (MMSE) scores ≥ 27. Pathological z-score values were defined as z < − 1.96 for FDG and z > + 1.96 for PiB.

### PET image processing and analysis

For each modality and each brother, as well as for the group of noncarriers, all PET images were realigned and spatially normalized into a common Montreal Neurological Institute space using a PET template (provided with SPM8 software) for FDG-PET and a population-specific PiB template [42] for PiB-PET images.

A gray matter mask was applied to a simplified probabilistic atlas [43] consisting of 12 bilateral ROIs. This atlas was then used for regional quantification of the PET tracers’ uptake, expressed in standardized uptake value ratio (SUVr) units with the pons as a reference region because this region was found to be a reliable reference for metabolism [44] and amyloid-β quantification [45] in both sporadic and familial Alzheimer’s disease. For comparison, all PET quantification analyses were repeated using the cerebellar gray matter as reference.

For both brothers, the narrow field of view (100 mm) of the respective first two FDG scans excluded some upper parts of the brain from the acquisitions. For each brother, and to keep their respective measurements at the different time points comparable, an individual mask was created that contained only voxels present in every successive FDG scan; the same mask was applied to the control group of noncarriers prior to calculating z-score values for regional FDG-PET uptake for each brother. All processing steps were performed using SPM8 on MATLAB (MathWorks, Natick, MA, USA).

### MRI acquisition

An MRI scan of the brain was obtained from both brothers in 2009, 14 years after baseline, when the brothers were 58 (A) and 57 (B) years old. This was the only MRI scan for brother B because he experiences claustrophobia, restricting the MRI analysis to being cross-sectional, even though more MRI scans were available from brother A. Structural MRI measurements for brothers A and B were expressed with z-scores using a control group of 14 cognitively normal noncarriers who had previously undergone structural MRI. The group of noncarriers had a median age of 59 years (range 42–73); 5 of the 14 were female, and all had MMSE scores ≥27.

The MRI datasets were acquired using a MAGNETOM Trio whole-body clinical 3-T MRI scanner (Siemens) equipped with a 12-channel phase-array head coil. All participants underwent the same MRI protocol. A high-resolution 3D T1-weighted magnetization-prepared rapid gradient-echo sequence image was acquired in sagittal plane (repetition time/echo time = 1780/3.42 milliseconds, inversion time = 900 milliseconds, 192 sagittal slices, voxel size 1 × 1 × 1 mm^3^, flip angle = 9 degrees). Full brain and skull coverage was required for the MRI datasets, and detailed quality control was carried out on all images according to previously published quality control criteria [46].

### MRI data processing, automated analysis, and visual rating

Cortical reconstruction and volumetric segmentation were performed using the FreeSurfer 5.1.0 image analysis suite (http://surfer.nmr.mgh.harvard.edu/), including removal of nonbrain tissue [47], intensity normalization [48], tessellation of the boundary between gray and white matter, surface deformation following intensity gradients to optimally place the gray/white and gray/CSF borders at the location where the greatest shift in intensity defines the transition to the other tissue class [49, 50], registration to a spherical atlas [51], and creation of a variety of regional cortical and subcortical data. Results were visually inspected and manually edited if necessary in order to ensure the accuracy of registration, skull stripping, segmentation, and cortical surface reconstruction.

After image processing, the volumetric measures for the left and right hippocampi were selected for analysis, normalized by the subject’s total intracranial volume [52]. Complementarily, the available longitudinal MRI data were clinically rated by an experienced neuroradiologist using the medial temporal atrophy (MTA) scale [53] on coronal reconstructions of the T1 sequence. Briefly, the degree of atrophy is scored from 0 (no atrophy) to 4 (end-stage degree of atrophy) in the hippocampus, parahippocampal gyrus, entorhinal cortex, and surrounding CSF spaces. The scores were then interpreted using age-adjusted cutoffs as detailed elsewhere [54].

### Statistical analysis

Regression models were used to fit the longitudinal trajectories of each of 12 neuropsychological tests (expressed as z-scores) separately for brothers A and B using age and age-squared as independent predictors, based on a previous FAD study in which age and age-squared were found to be significant predictors of cognition [55]. The statistical significance level was set at *P* < 0.05 to determine decline over time. The Bonferroni multiple-comparisons correction was applied to account for the 12 repeated tests. Significant results are reported both before and after Bonferroni correction.

## Results

### Clinical characteristics

#### Brother A

Brother A was first included in the study in 1995 at 44 years of age, at which point he had no subjective cognitive complaints. He had 10 years of formal education and was employed as a welder. He had a history of asthma and migraine but was otherwise healthy, a nonsmoker, did not consume alcohol, and was taking no medication.

In 2006 (at the age of 55), brother A was experiencing depression and anxiety and had sleep disturbances. He also described his work as stressful. He scored 27/30 points on the MMSE, and an objective decline in episodic memory tests compared with baseline was noted (*see* Additional file 1). Thus, he received a diagnosis of MCI because his activities of daily living were preserved. He was also diagnosed with mild depression and received a prescription for citalopram. He took the medication for 1 month but then decided to discontinue it because he did not experience any positive effect.

One year later, at the age of 56 (5 years after the mean family-specific age of symptom onset), brother A had experienced a rapid decline in cognitive function and had been dismissed from his job. He reported a severe impairment in episodic memory and also had problems with executive functioning and visuospatial abilities. In addition, he was experiencing increasing apraxia. His MMSE score was 22/30, but no further neuropsychological assessment was done at that time. He received a diagnosis of dementia due to Alzheimer’s disease, and treatment with galantamine was initiated.

At the age of 57, brother A had somewhat improved cognitively and felt that the medication had a positive effect on his memory. His MMSE score was higher than the year before (24/30). In 2009, at the age of 58, worsening of his symptoms had been fast. He had stopped driving and was losing weight rapidly because he often forgot to eat. His MMSE score was 11/30, and severe memory deficits were apparent during a standard interview.

In 2013, at the age of 62, brother A was in a nursing home and in the final stage of Alzheimer’s disease requiring around-the-clock assistance, despite still being quite mobile. At this time, it was no longer possible to assess him cognitively with the MMSE or other neuropsychological tests. A final study visit to the nursing home was made in the spring of 2015, when the patient was confined to a wheelchair. He died in the fall of 2015 at the age of 64, 9 years after receiving his MCI diagnosis and 13 years after the mean family-specific symptom onset.

An autopsy performed on the brain of brother A revealed extensive Alzheimer’s pathology, fulfilling the CERAD (Consortium to Establish a Registry for Alzheimer’s Disease) criteria for definitive Alzheimer’s disease [56]. His Braak stage was V–VI [57], and cerebral amyloid angiopathy was present.

#### Brother B

Brother B was 43 years old when he first participated in the study in 1995. He had 9 years of formal education and worked in shifts at a factory where metal objects receive surface coating. The work used to involve heavy lifting, but in recent years he had mostly been overseeing an automated process. He had high blood pressure and obstructive sleep apnea, as well as chronic musculoskeletal pain in the head and neck, back, hands, and knees. The pain was judged to be related to osteoarthritis and hard labor, and he medicated regularly with glucosamine, ibuprofen, and paracetamol. He also took losartan for his high blood pressure. In addition, he had received sleep treatment with a continuous positive airway pressure (CPAP) machine over a period of a few years at the most, but he had stopped using the device because he felt it was not alleviating his symptoms of fatigue. He had never smoked and did not consume alcohol. At inclusion, he had no subjective cognitive complaints.

In 2006, at the age of 54, he had an MMSE score of 29/30 and no signs of cognitive decline on a comprehensive neuropsychological assessment. In 2009, at the age of 57, his MMSE score was still 29, and in 2010, it was 27. During this period, he described short and interrupted sleep with pronounced daytime fatigue. At the age of 59 (in 2011), he was experiencing subjective impairment in episodic memory and concentration. He felt depressed, constantly tired, had frequent headaches, and was under a lot of stress in the workplace. At this point, he had stopped using the CPAP machine, and his MMSE score was 30. At a study visit 4 years later, in 2015, brother B (at the age of 63) was still employed and was independent in all activities of daily living. He did not report any subjective cognitive complaints at this time, and this was confirmed by close relatives. During 2016, he was on sick leave because of a rheumatological disease unrelated to the osteoarthritis described above. At a final study visit in 2017 (at the age of 65), his predominant leisure activity was playing chess (a lifelong activity as an adult), and there was no change in his ability to play chess or perform other activities. He was still working full-time and was capable of performing all necessary household chores.

### Neuropsychological assessments

Regression analyses for brother A, with each cognitive test score as a dependent variable (longitudinal repeated measures) and age and age-squared as independent predictors, were significant in 9 of 12 tests (4 tests after Bonferroni correction) and well-fitted (multiple *r*^2^ ranged between 0.62 and 0.99) to a curvilinear decline (age was a significant predictor in 10 tests, and age-squared was a significant predictor in 6 tests). The regression coefficients for age and age-squared were always negative. In brother B, the age-related linear regression was negative for the Rey-Osterrieth copy test and positive for the RAVL learning test; however, none of the regression models were significant for any test after Bonferroni correction.

The comparison between brothers across time is exemplified in Additional file 1, showing marked decline for brother A compared with invariant performance for brother B in episodic memory. It is worth noting that both brothers had similar values for estimated premorbid cognitive function [37], which were z = + 0.42 (brother A) and z = + 0.19 (brother B). The current (at inclusion) values for global cognitive function were z = + 0.39 and z = − 0.13, respectively, indicating that both brothers had normal and preserved global cognitive function at inclusion. In Table 2, cognitive test results across all assessments compared with the reference group are presented for three tests having demonstrated high sensitivity for cognitive decline in FAD (Rey Auditory Verbal Learning, Block Design, and Digit Symbol [55]). Interestingly, the pattern of test results showed a continuous decline across the years from baseline until endpoint in brother A (> 4 SD on Rey Auditory Verbal Learning and > 3 SD on Block Design and Digit Symbol), in contrast to brother B (< 0.5 SD).

**Table 2:** Longitudinal neuropsychological test results for brothers A and B

	Year	Brother	RAVL	Block design	Digit symbol
Baseline	1995	A	+ 0.74	+ 1.48	+ 1.41
+ 2 years	1997	A	+ 0.45	+ 2.00	+ 1.06
+ 6 years	2001	A	+ 0.16	+ 1.09	+ 0.97
+ 11 years	2006	A	**−1.57**	+ 0.70	+ 0.37
+ 13 years	2008	A	**−2.91**	**−1.64**	−1.35
+ 14 years	2009	A	**−3.30**	**−1.90**	**−2.56**
Baseline	1995	B	−0.32	+ 1.48	+ 0.80
+ 2 years	1997	B	−0.61	+ 2.00	+ 0.54
+ 6 years	2001	B	−0.22	+ 0.57	+ 0.11
+ 11 years	2006	B	−0.41	+ 0.83	+ 0.63
+ 14 years	2009	B	−0.32	+ 1.48	+ 0.46
+ 16 years	2011	B	−0.32	+ 1.35	+ 0.57
+ 18 years	2013	B	+ 0.16	+ 0.57	+ 0.11
+ 22 years	2017	B	+ 0.16	+ 0.96	+ 0.28

*RAVL* Rey Auditory Verbal Learning

The brothers underwent repeated neuropsychological testing between 1995 and 2017. Numbers in bold are test scores with z less than − 1.5, representing results below the cognitively normal threshold

### CSF biomarkers

The brothers underwent a lumbar puncture in 1995 (at the ages of 43 and 44) and again in 2006 (at the ages of 54 and 55). The three CSF biomarkers currently in clinical use—amyloid-β_42_, T-tau, and P-tau—were measured in 2012, and the results are presented in Table 3. In the samples from 1995, brother A had reduced levels of CSF amyloid-β_42_ (321 ng/L), whereas all other CSF biomarkers in both brothers were within reference ranges. In the samples from 2006, brother B still had all three biomarkers within the normal reference range (Table 3), whereas both T-tau (704 ng/L) and P-tau (82 ng/L) were elevated in brother A. Interestingly, amyloid-β_42_ was no longer decreased in brother A in the sample from 2006.

**Table 3:** Biomarker levels in the cerebrospinal fluid of brothers A and B

		Amyloid-β_42_ (ng/L) Analyzed in 2012	Amyloid-β_42_ (ng/L) Analyzed in 2017	T-tau (ng/L)	P-tau (ng/L)
1995	Brother A (age 44 yr)	**321**	**453**	156	37
	Brother B (age 43 yr)	652	N.A.	136	26
2006	Brother A (age 55 yr)	730	**421**	**704**	**82**
	Brother B (age 54 yr)	1914	1256	212	41

*Abbreviations: CSF* Cerebrospinal fluid, *N.A.* Not available, *P-tau* Phosphorylated tau, *T-tau* Total tau

Biomarker levels that fall outside the reference range are highlighted in bold. The normal values for these three biomarkers applied at the Clinical Neurochemistry Laboratory at the Sahlgrenska University Hospital in Mölndal, Sweden, were used as a reference. At the laboratory in Mölndal, the normal reference level for CSF amyloid-β_42_ is > 550 ng/L. The reference level for CSF T-tau is < 300 ng/L for individuals between 18 and 45 years of age and < 400 ng/L for those who are over 45 years old. CSF P-tau should be < 60 ng/L for those under 60 years of age and < 80 ng/L for those 60 years or older

CSF amyloid-β_42_ was reanalyzed in 2017 because the levels of amyloid-β_42_ were unusually high in the sample from 2006 in brother B, and even in brother A, after taking into account the clinical history and the low amyloid-β_42_ levels in 1995. Unfortunately, there was not enough sample volume to reanalyze the sample from brother B from 1995; however, the other three samples were reanalyzed (brother A from 1995 and 2006 and brother B from 2006). Interestingly, brother A still had low amyloid-β_42_ levels in the sample from 1995 according to the reanalysis (453 ng/L) and now also in the sample from 2006 (421 ng/L), contrary to what was observed in the original analysis in 2012. The amyloid-β_42_ levels in the sample from brother B from 2006 were still high in 2017 (1256 ng/L), albeit not as high as originally observed.

### PET imaging

**Fig. 1:**
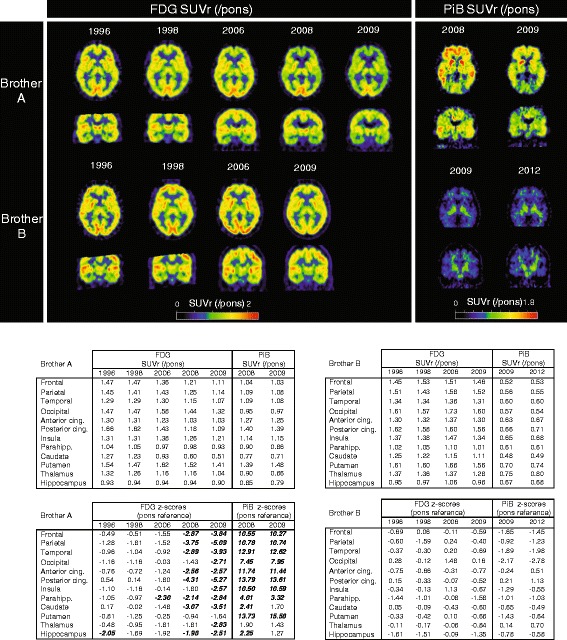
Longitudinal FDG and PiB positron emission tomographic (PET) scans for brothers A and B with corresponding uptake values in SUVr(/pons) and z-scores. The two upper rows of the figure represent the longitudinal FDG and PiB PET scans for brother A during repeated follow-up examinations. The year of each examination is noted at the top of each column. The lower two rows of the figure represent the corresponding longitudinal FDG and PiB PET scans for brother B. The values included in the tables are standardized uptake value ratios (SUVr) for the ROIs in the study, with the pons used as a reference region, as well as the corresponding z-score values with respect to the control group of noncarriers. FDG z-score values less than − 1.96 and PiB z-score values greater than + 1.96 are indicated in bold italic type. *FDG* [^18^F]fluorodeoxyglucose, *GM* Gray matter, *PiB* [^11^C]Pittsburgh compound B, *SUVr* Standardized uptake value ratio

Longitudinal FDG and PiB scans for both brothers are shown in Fig. 1, including uptake values in SUVr(/pons) evaluated in 12 ROIs as well as in z-score units relative to the control group. Additional file 2 includes respective PET uptake values using the cerebellar gray matter as a reference. Additional file 3 illustrates the longitudinal regional FDG and PiB uptake of both brothers in comparison with the control group of noncarriers.

In 2006, when brother A received a diagnosis of MCI at the age of 55, there was low FDG uptake in the parahippocampus (as measured by z-scores) but normal uptake in other ROIs (Fig. 1; pons used as a reference). Two years later, 1 year after developing dementia due to Alzheimer’s disease, brother A had decreased FDG uptake in all ROIs except the occipital cortex, insula, putamen, and thalamus (Fig. 1). One year later, in 2009, at the age of 58, the only region with normal FDG uptake in brother A was the putamen. A similar pattern of declining FDG uptake was noted for brother A when the cerebellar gray matter was used as a reference (Additional file 2). Conversely, brother B had normal FDG uptake in all ROIs across time when the pons was used as a reference (Fig. 1); normal FDG uptake was also observed when the cerebellar gray matter was used as a reference, except for a slightly reduced hippocampal FDG uptake on the last two successive scans, without signs of progression (Additional file 2).

PiB uptake for brother A was high (as measured by z-scores) in all brain regions except the thalamus in 2008 at the age of 57 and in all regions except the thalamus, caudate nucleus, and hippocampus in 2009 at the age of 58, when the pons was used as a reference (Fig. 1); similar results were observed when the cerebellar gray matter was used as a reference (Additional file 2), and PiB uptake was above the cutoff for amyloid-β positivity [42] of SUVr = 1.41 in most brain regions, in both 2008 and 2009. In contrast, there was no elevated PiB uptake in brother B (as measured by z-scores) in either 2009 or 2012, at 57 and 60 years old, respectively (Fig 1; pons used as a reference); any observed difference in PiB retention between 2009 and 2012 can be considered within the test-retest variability [58]. Interestingly, though, the PiB uptake in brother B was slightly low (as measured by z-scores) in the occipital cortex in 2009 at the age of 57 and in the occipital and temporal cortices in 2012 at 60 years old (Fig. 1; pons used as a reference). When the cerebellar gray matter was used as reference, cortical PiB uptake was below the cutoff for amyloid-β positivity of SUVr = 1.41 in all brain regions, except for slightly elevated z-scores in the posterior cingulate cortex and the thalamus in 2009, and in both regions plus the anterior cingulate cortex in 2012 (Additional file 2), which were within normal values, however, when the pons was used as the reference region (Fig. 1). Overall, and in sharp contrast to Brother A, Brother B had FDG and PiB uptake values that were within control group values, as illustrated in Additional file 3.

### Brain MRI

**Fig. 2:**
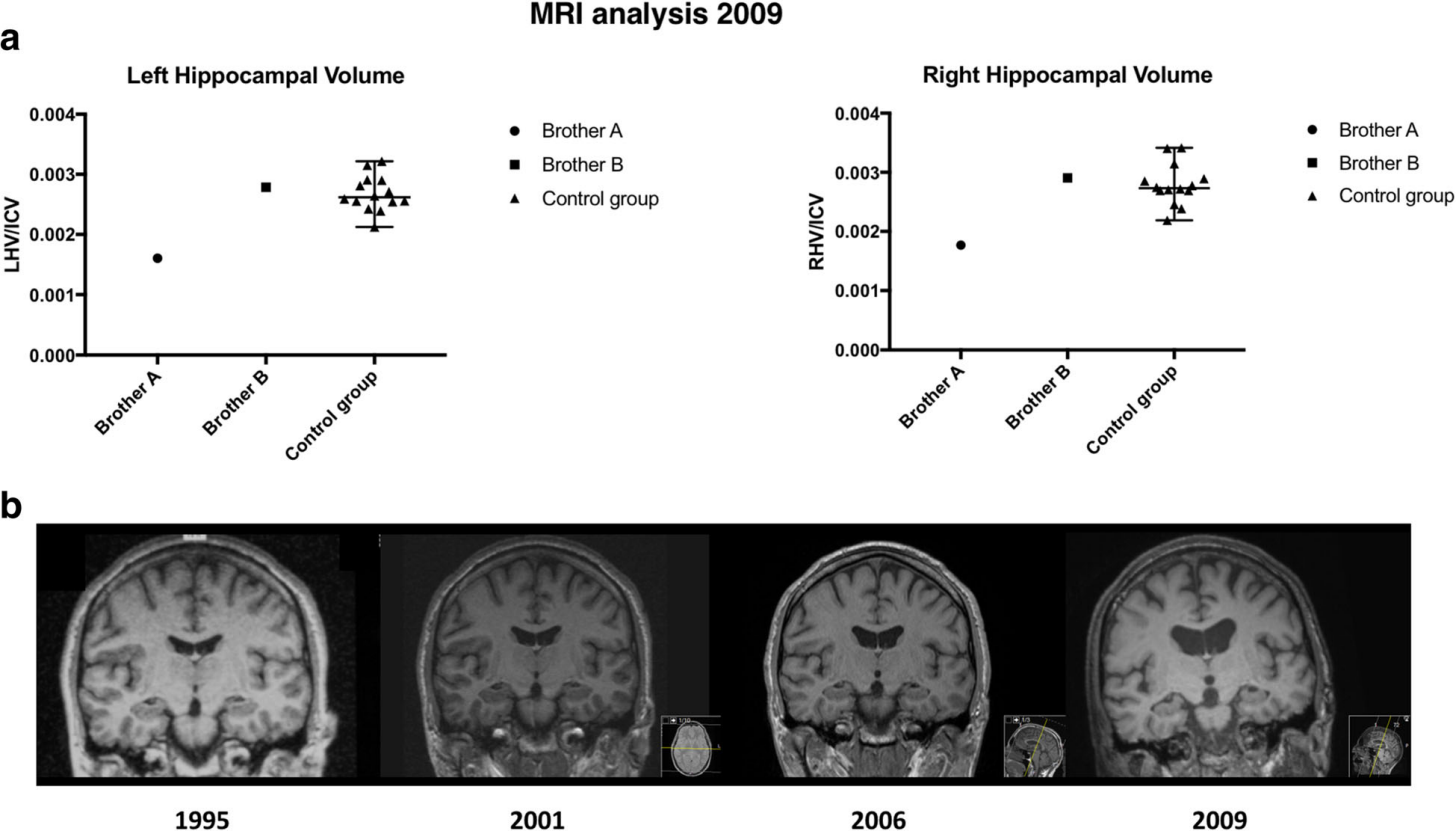
Hippocampal volumes of brothers A and B on magnetic resonance imaging (MRI) scans. **a** Cross-sectional data on the volumes of the left (LHV) and right (RHV) hippocampi of brothers A and B on MRI scans from 2009, when the brothers were 58 and 57 years old, respectively. At this time point, brother A had had an Alzheimer’s disease diagnosis for 2 years. The control group consists of 14 noncarriers without cognitive symptoms. The volumes (in mm^3^) have been divided by the intracranial volume (ICV) of each subject to correct for differences in head size. **b** Coronal MRI scans of brother A from 1995 (at the age of 44), 2001 (at the age of 50), 2006 (at the age of 55), and 2009 (at the age of 58)

The left and right hippocampal volumes of both brothers are presented in Fig. 2, along with those values for the control group of noncarriers. Both left and right hippocampal volumes of brother A fell below the range of the control group (z = − 2.9 and − 3.6; left and right values, respectively), whereas the hippocampal volumes of brother B fell within the control group range (z = + 0.3 for both left and right hippocampi) (*see* Fig. 2).

The MRI analysis above is restricted to being cross-sectional because brother B had only one MRI scan (in 2009 at the age of 57) owing to claustrophobia. The earlier MRI scans available from brother A were visually inspected by an experienced neuroradiologist and assessed for the MTA score. The MTA score was estimated to be zero (no atrophy) bilaterally in the scans from 1995, 2001, and 2006 (at the ages of 44, 50, and 55, respectively); the scan from 1997 was not available for analysis. In the scan from 2009, the MTA scores were 1 on the right side and 2 on the left side (Fig. 2); the age-adjusted cutoff for abnormality is 1.5 from averaged left/right scores [54].

## Discussion

Autosomal dominant mutations in the *PSEN1* gene leading to FAD are currently considered to be almost fully penetrant. This study on two brothers born 1 year apart and both carrying the *PSEN1* H163Y mutation presents the first biomarker-verified case of reduced penetrance of a *PSEN1* mutation. The brothers were followed with repeated clinical evaluations over a period of 22 years, during which time the older brother, referred to as brother A, developed Alzheimer’s disease with typical progressive cognitive decline. The cognitive test results revealed a gradual decline, first in episodic memory, visuospatial ability, and executive function, then followed by deterioration in all examined cognitive domains. In contrast, no such decline was apparent in his brother, referred to as brother B. Brother A showed typical biomarker signs of Alzheimer’s disease, starting with an early decrease in CSF amyloid-β_42_ and later progressing to an increase in CSF T-tau and P-tau. Interestingly, however, the level of CSF amyloid-β_42_ of brother A returned to normal as the disease progressed. Owing to this unusual development of amyloid-β_42_ levels, we reanalyzed amyloid-β_42_ in the CSF samples from brother A and also in one of the samples from brother B. The amyloid-β_42_ levels in the reanalysis were lower than in the original analysis in all three samples, with brother A having pathological amyloid-β_42_ levels on both sampling occasions. Unfortunately, we do not have a solid explanation for this discrepancy, but it suggests that these results should be interpreted with caution. Brother A had decreased volumes of both left and right hippocampi on MRI and decreased glucose metabolism on FDG-PET observed prior to atrophy in several brain regions, including parietal and temporal areas. He also had cerebral amyloidosis observed on PiB-PET. Brother A died at the age of 64, 9 years after being diagnosed with MCI. On autopsy, typical neuropathological signs of Alzheimer’s disease were present.

In contrast, brother B showed no signs of cognitive decline on neuropsychological assessment during the follow-up period. The mean age of onset of the first cognitive symptoms in this family is 51 ± 7 years, and the final evaluation of brother B was performed in 2017 when he was age 65, 14 years past the mean age of onset. Furthermore, brother B had normal levels of CSF biomarkers at the age of 54 and did not show signs of hippocampal atrophy on MRI at the age of 57. Brother B had overall no pathological changes on FDG-PET and PiB-PET, the most recent being performed at the ages of 57 and 60, respectively.

According to the current understanding of the pathological processes in Alzheimer’s disease, amyloid-β biomarkers in CSF and on PiB-PET first become abnormal years or even decades before the onset of the first cognitive symptoms; biomarkers of neurodegeneration, including CSF T-tau, FDG-PET, and volumetric MRI, are thought to become abnormal later in the disease process and correlate better with the severity of symptoms [59]. Therefore, it is especially interesting that brother B had normal levels of CSF amyloid-β_42_ at the age of 54, 3 years past the mean age of symptom onset in his family, and no abnormal uptake on PiB-PET at the age of 60, 9 years past the mean symptom onset age. Currently, an individual is considered to be in the preclinical stage of Alzheimer’s disease when there is evidence of either amyloid-β pathology [60] or both amyloid-β and tau pathology [61], but no cognitive symptoms are present. According to this definition of preclinical Alzheimer’s disease, brother B still had not reached the stage of preclinical Alzheimer’s disease at the age of 60, when he had his last PiB-PET scan.

It is interesting to note that in our recent study done in Sweden, where carriers of FAD mutations (including the *PSEN1* H163Y mutation) were followed with repeated neuropsychological testing over the course of four decades, a decline in episodic memory, visuospatial ability, and executive function was observed several years before the mean family-specific onset of symptoms [55]. No such decline was observed in brother B, with the most recent neuropsychological evaluation being performed in 2017 (at the age of 65), which renders support for the assumption that he had not reached the earliest clinical stages of Alzheimer’s disease. In addition, in a previous longitudinal FDG-PET imaging study in six presymptomatic members of the same *PSEN1* H163Y family [33], researchers reported an early decline in glucose metabolism in the thalamus several years before the expected clinical onset, as well as temporal decline in glucose metabolism with respect to years until expected clinical onset in mutation carriers. No such decline in FDG-PET was observed in the longitudinal study of brother B, providing biomarker evidence that he was not near the clinical onset of the disease. Overall, the combined evidence from the biomarker data and cognition presented in this study strongly suggests that the *PSEN1* H163Y mutation had reduced penetrance in brother B. Given that genotyping was performed repeatedly on multiple samples collected on separate occasions and from different tissues, we are convinced that brother B is a true mutation carrier.

A few cases of reduced penetrance of *PSEN1* mutations have been described previously [21–24]. Neither biomarker results nor neuropsychological assessments were available in these cases, but one subject with reduced penetrance had a normal MMSE score at the age of 68 (with the median age of onset in the family being 55) [21]. Also, some *PSEN1* mutations have been reported to have a wide range of ages at symptom onset [22–24] as well as wide clinical phenotypic variation [10]. Age at symptom onset reported in the literature is often based on anecdotal data collected retrospectively, and in such settings, cognitive decline might go unnoticed for some time. The age of onset of first symptoms could therefore be lower than generally reported. This does not apply in the case of brother B, owing to the rigorous prospective follow-up over two decades, which supports the existence of reduced penetrance of this particular *PSEN1* mutation.

A recent study on the *PSEN1* E280A pedigree, the largest known FAD pedigree globally, revealed that the onset of FAD symptoms in *PSEN1* E280A mutation carriers carrying the *APOE* ε2 allele was, on average, 8.24 years later than in those not carrying the ε2 allele. In that study, the *APOE* ε4 allele did not have a significant effect on symptom onset [62]. Authors of a recent meta-analysis of age of symptom onset in FAD families reported that ε4 allele carriers had earlier onset and ε2 carriers a later onset; however, these findings did not reach statistical significance or add to the explanation of the variance in age of symptom onset [9]. These findings corroborate previous findings of lack of effect of the ε4 allele in *PSEN1* mutations [63]. With regard to the brothers in the present study, brother A carried the ε2/ε4 genotype, whereas brother B carries the ε2/ε3 genotype. Because both brothers carry an ε2 allele, one could assume that the differences observed in age at symptom onset are not likely explained by differences in their APOE genotype. However, the possibility that the combination of an ε2 allele and an ε3 allele is protective in brother B cannot be entirely excluded.

## Conclusions

The present study strongly suggests that the *PSEN1* H163Y mutation has a reduced penetrance in brother B. This is supported by a longitudinal follow-up of the subject over 22 years, starting at the age of 43 (8 years before expected symptom onset) and ending at the age of 65 (14 years past the expected symptom onset), and his remaining amyloid-β biomarker-negative until the last time point with available biomarker assessment (age 60 years) and showing no cognitive decline on neuropsychological tests at the age of 65. These findings have implications for genetic counseling because one cannot assume that *PSEN1* mutations are 100% penetrant and that family-specific mean age of symptom onset has greater variability than previously reported. The findings are also hypothesis-generating because they suggest the presence of a factor or factors in this individual that can be disease-modifying. Whether these factors are genetic, epigenetic, or environmental remains a subject of further study.

## Additional files

Additional file 1: Longitudinal z-scores for the RAVL total learning test measuring episodic memory in brothers A and B. The scatterplot shows episodic memory as evaluated by the RAVL total learning test and expressed in z-score values versus years to the expected clinical onset of Alzheimer’s disease. The longitudinal trajectories are illustrated by LOESS curves for brothers A and B. *RAVL* Rey Auditory Verbal Learning. (PDF 222 kb)

Additional file 2: Longitudinal FDG and PiB PET scans for brothers A and B, and corresponding uptake values in SUVr(/cerebellar gray matter) and z-scores. The two upper rows of the figure represent the longitudinal FDG and PiB PET scans for brother A during repeated follow-up examinations. The year of each examination is noted at the top of each column. The lower two rows of the figure represent the corresponding longitudinal FDG and PiB PET scans for brother B. The values included in the tables are standardized uptake value ratios (SUVr) for the ROIs in the study, with the cerebellar gray matter used as a reference region, as well as the corresponding z-score values with respect to the control group of noncarriers. FDG z-score values less than − 1.96 and PiB z-score values greater than + 1.96 are indicated in bold italic type. *FDG* [^18^F]fluorodeoxyglucose, *GM* Gray matter, *PiB* [^11^C]Pittsburgh compound B, *SUVr* Standardized uptake value ratio. (PDF 1471 kb)

Additional file 3: Longitudinal FDG and PiB-PET uptake in SUVr(/pons and/cerebellar gray matter) units in brothers A and B compared with the control group of noncarriers in representative ROIs. *GM* Gray matter, *PCC* Posterior cingulate cortex, *SUVr* Standardized uptake value ratio, *Temp* Temporal. (PDF 125 kb)

## Abbreviations

*APP*: Amyloid precursor protein gene
cDNA: Complementary DNA
CPAP: Continuous positive airway pressure
CSF: Cerebrospinal fluid
ELISA: Enzyme-linked immunosorbent assay
FAD: Familial Alzheimer’s disease
FDG: [^18^F]fluorodeoxyglucose
MCI: Mild cognitive impairment
MMSE: Mini Mental State Examination
MTA: Medial temporal atrophy
PET: Positron emission tomography
PiB: [^11^C]Pittsburgh compound B
*PSEN1*: Presenilin 1 gene
*PSEN2*: Presenilin 2 gene
P-tau: Phosphorylated tau (tau phosphorylated at threonine 181)
RAVL: Rey Auditory Verbal Learning
SNP: Single-nucleotide polymorphism
SUVr: Standardized uptake value ratio
T-tau: Total tau
