# Supplementary figures and images for: Reduced penetrance of the PSEN1 H163Y autosomal dominant Alzheimer mutation: a 22-year follow-up study
Source: Alzheimers Res Ther. 2018 May 10;10:45. doi: 10.1186/s13195-018-0374-y (PMC5944151; doi:10.1186/s13195-018-0374-y)

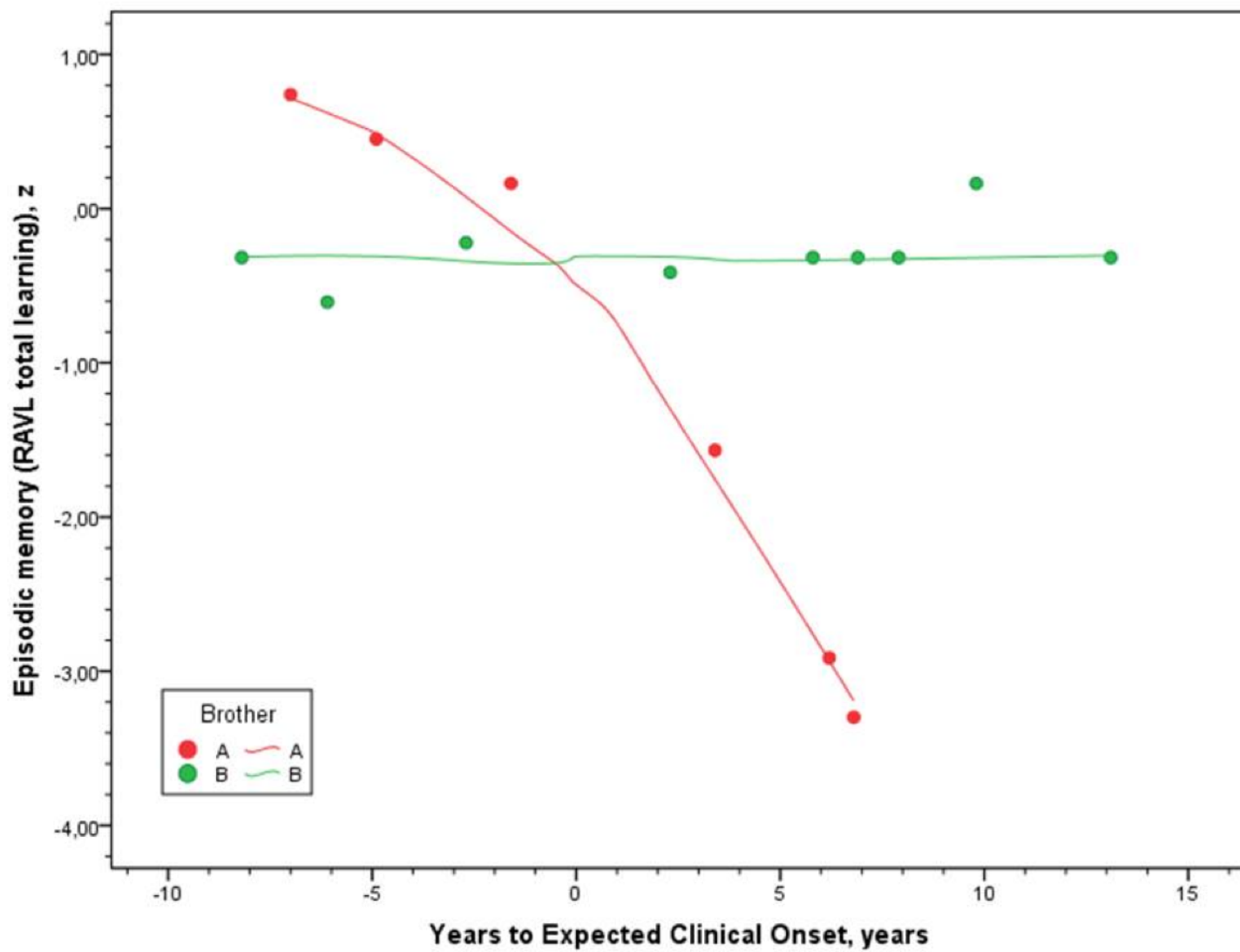

Supplement: Supplementary file 1 — Longitudinal z-scores for the RAVL total learning test measuring episodic memory in brothers A and B. The scatterplot shows episodic memory as evaluated by the RAVL total learning test and expressed in z-score values versus years to the expected clinical onset of Alzheimer’s disease. The longitudinal trajectories are illustrated by LOESS curves for brothers A and B. RAVL Rey Auditory Verbal Learning. (PDF 222 kb) [file 13195_2018_374_MOESM1_ESM.pdf]

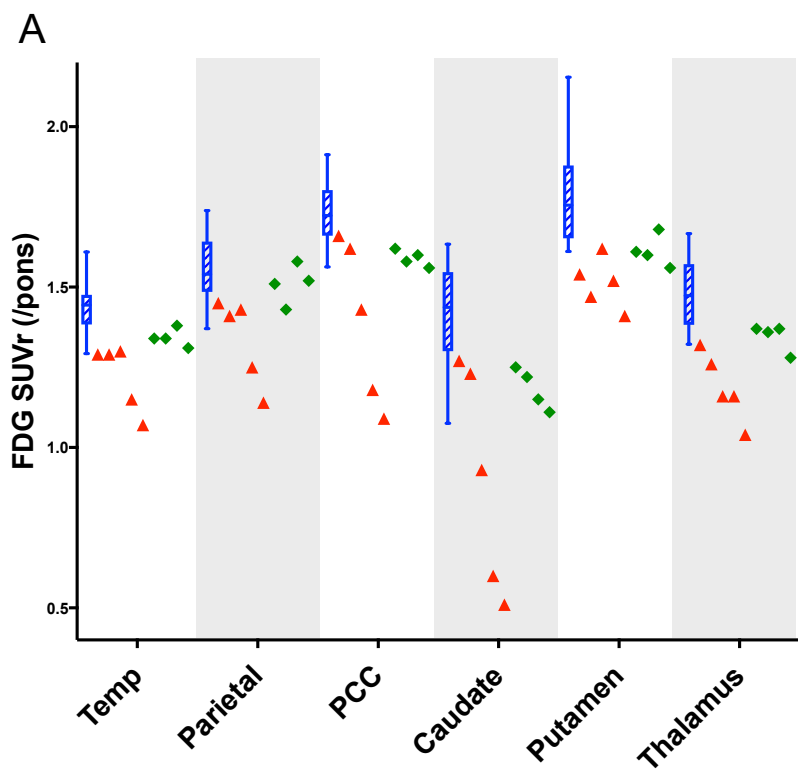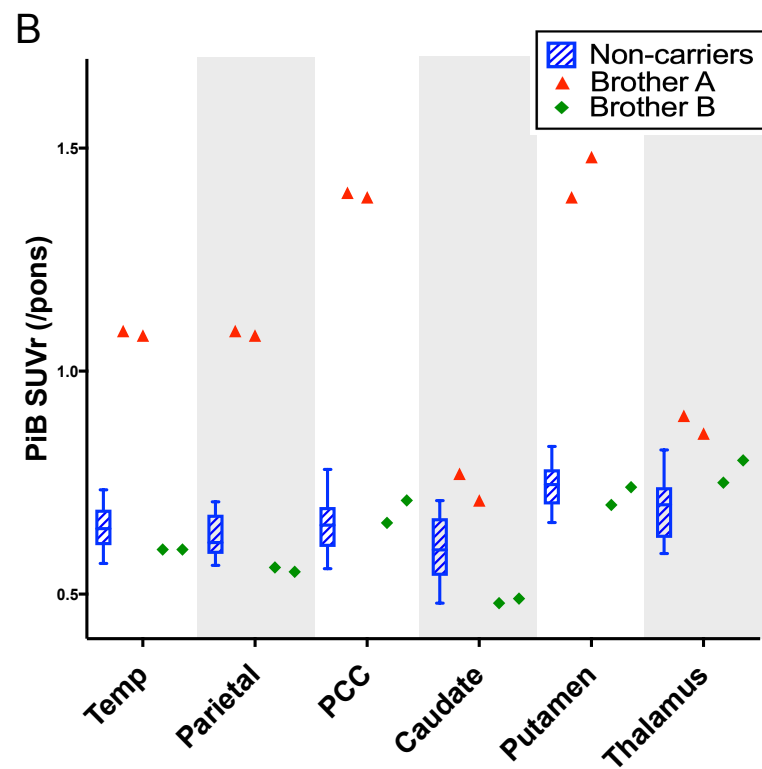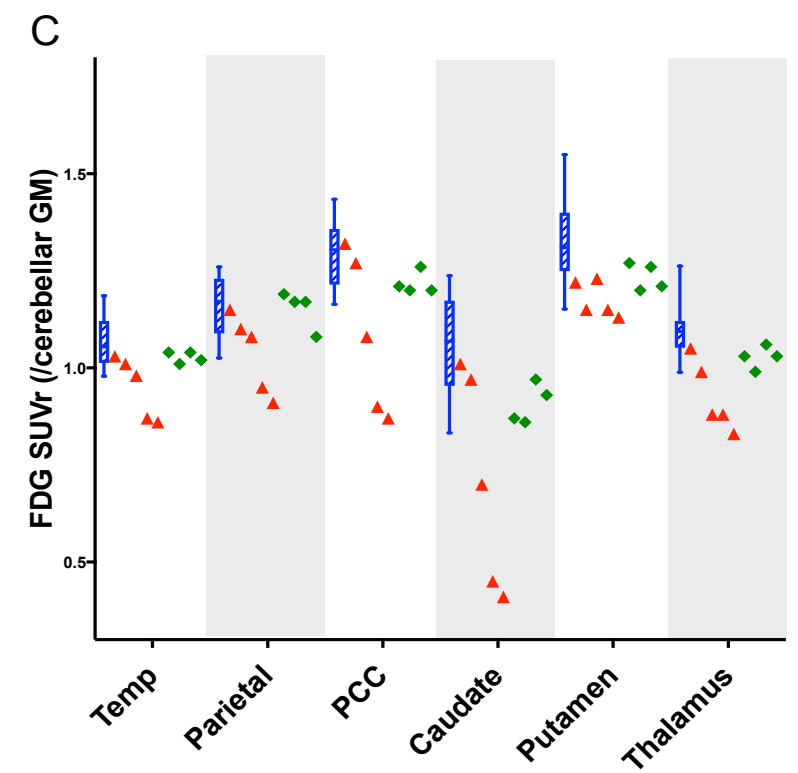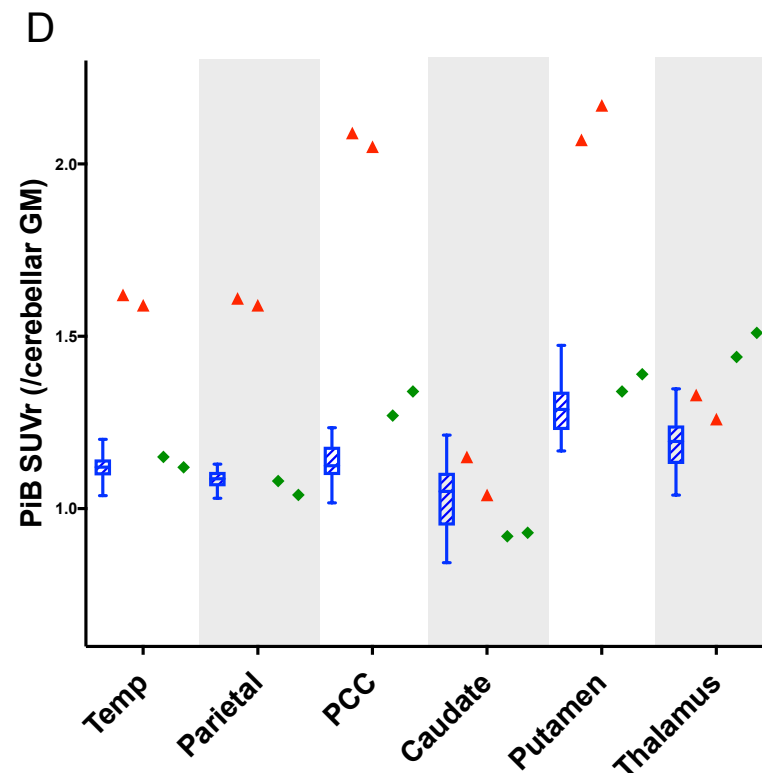

Supplement: Supplementary file 3 — Longitudinal FDG and PiB-PET uptake in SUVr(/pons and/cerebellar gray matter) units in brothers A and B compared with the control group of noncarriers in representative ROIs. GM Gray matter, PCC Posterior cingulate cortex, SUVr Standardized uptake value ratio, Temp Temporal. (PDF 125 kb) [file 13195_2018_374_MOESM3_ESM.pdf]
