# Supplementary material for: Reduced penetrance of the PSEN1 H163Y autosomal dominant Alzheimer mutation: a 22-year follow-up study
Source: Alzheimers Res Ther. 2018 May 10;10:45. doi: 10.1186/s13195-018-0374-y (PMC5944151; doi:10.1186/s13195-018-0374-y)
Supplement: Supplementary file 2 — Longitudinal FDG and PiB PET scans for brothers A and B, and corresponding uptake values in SUVr(/cerebellar gray matter) and z-scores. The two upper rows of the figure represent the longitudinal FDG and PiB PET scans for brother A during repeated follow-up examinations. The year of each examination is noted at the top of each column. The lower two rows of the figure represent the corresponding longitudinal FDG and PiB PET scans for brother B. The values included in the tables are standardized uptake value ratios (SUVr) for the ROIs in the study, with the cerebellar gray matter used as a reference region, as well as the corresponding z-score values with respect to the control group of noncarriers. FDG z-score values less than − 1.96 and PiB z-score values greater than + 1.96 are indicated in bold italic type. FDG [18F]fluorodeoxyglucose, GM Gray matter, PiB [11C]Pittsburgh compound B, SUVr Standardized uptake value ratio. (PDF 1471 kb) [file 13195_2018_374_MOESM2_ESM.pdf]

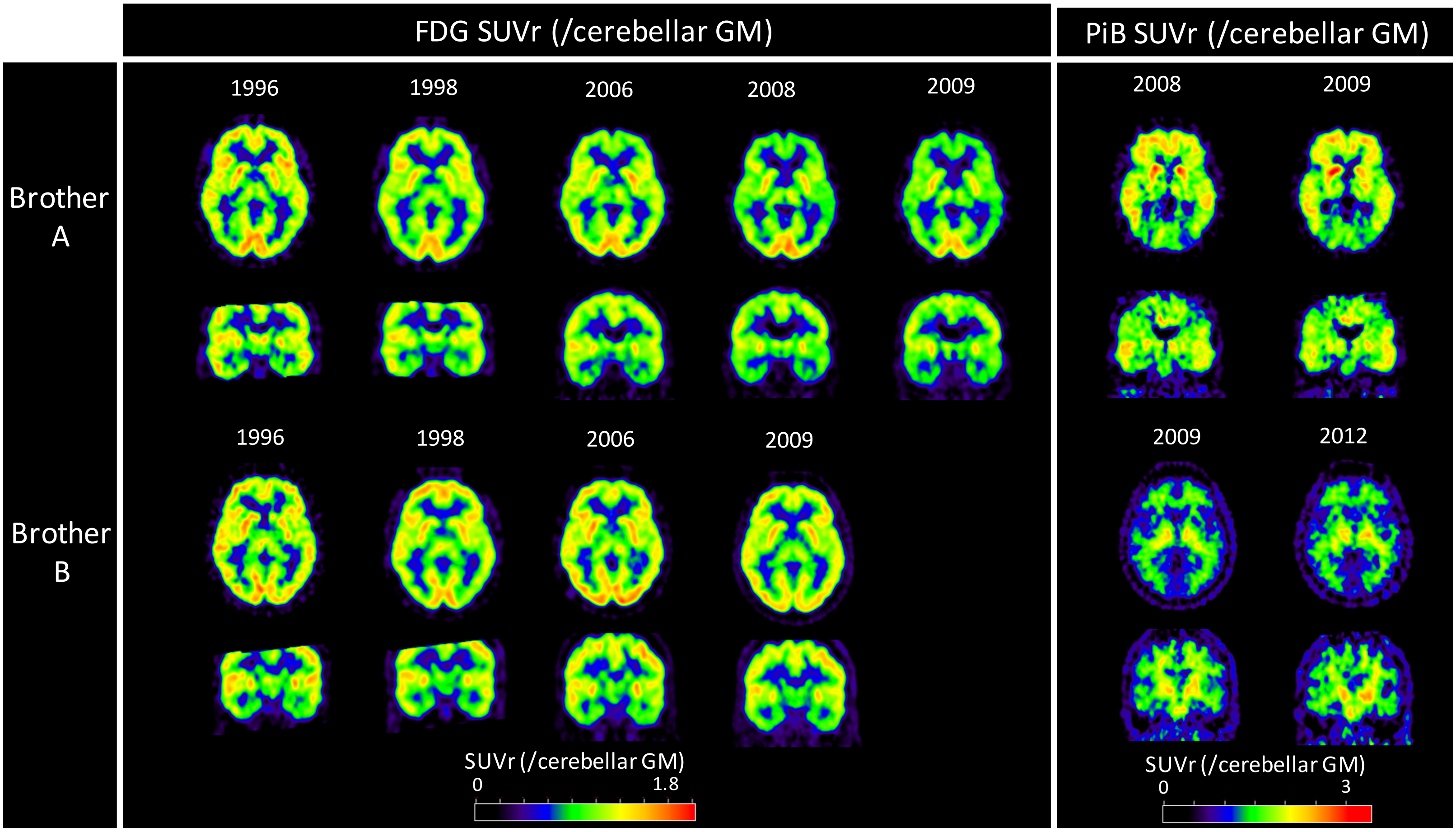

Brother A	FDG SUVr (/cerebellar GM)					PiB SUVr (/cerebellar GM)	
	1996	1998	2006	2008	2009	2008	2009
Frontal	1.17	1.15	1.02	0.92	0.89	1.55	1.52
Parietal	1.15	1.10	1.08	0.95	0.91	1.61	1.59
Temporal	1.03	1.01	0.98	0.87	0.86	1.62	1.59
Occipital	1.17	1.15	1.19	1.10	1.06	1.41	1.43
Anterior cing.	1.03	1.03	0.93	0.78	0.83	1.89	1.85
Posterior cing.	1.32	1.27	1.08	0.90	0.87	2.09	2.05
Insula	1.04	1.03	1.04	0.96	0.97	1.70	1.69
Parahipp.	0.83	0.82	0.73	0.74	0.75	1.34	1.26
Caudate	1.01	0.97	0.70	0.45	0.41	1.15	1.04
Putamen	1.22	1.15	1.23	1.15	1.13	2.07	2.17
Thalamus	1.05	0.99	0.88	0.88	0.83	1.33	1.26
Hippocampus	0.74	0.74	0.71	0.71	0.72	1.26	1.16

Brother B	FDG SUVr (/cerebellar GM)				PiB SUVr (/cerebellar GM)	
	1996	1998	2006	2009	2009	2012
Frontal	1.14	1.12	1.13	1.16	0.99	0.99
Parietal	1.19	1.17	1.17	1.08	1.08	1.04
Temporal	1.04	1.01	1.04	1.02	1.15	1.12
Occipital	1.30	1.23	1.26	1.19	1.08	1.02
Anterior cing.	1.03	1.00	1.02	1.00	1.21	1.27
Posterior cing.	1.21	1.20	1.26	1.20	1.27	1.34
Insula	1.11	1.04	1.06	1.05	1.24	1.28
Parahipp.	0.83	0.78	0.79	0.80	1.17	1.15
Caudate	0.87	0.86	0.97	0.93	0.92	0.93
Putamen	1.27	1.20	1.26	1.21	1.34	1.39
Thalamus	1.03	0.99	1.06	1.03	1.44	1.51
Hippocampus	0.80	0.75	0.74	0.73	1.29	1.29

Brother A	FDG z-scores (cerebellar GM reference)					PiB z-scores (cerebellar GM reference)	
	1996	1998	2006	2008	2009	2008	2009
Frontal	-0.12	-0.30	-1.66	***-2.77***	***-3.12***	***13.70***	***12.66***
Parietal	-0.80	-1.54	-1.87	***-3.90***	***-4.45***	***19.15***	***18.41***
Temporal	-0.49	-0.78	-1.27	***-3.03***	***-3.25***	***10.78***	***10.09***
Occipital	-0.78	-0.96	-0.40	-1.70	***-2.24***	***9.34***	***9.80***
Anterior cing.	-0.53	-0.60	-1.42	***-2.65***	***-2.29***	***12.96***	***12.21***
Posterior cing.	0.96	0.35	***-2.08***	***-4.39***	***-4.73***	***16.72***	***16.08***
Insula	-0.58	-0.86	-0.54	***-1.97***	-1.81	***11.09***	***10.81***
Parahipp.	-0.56	-0.68	***-2.44***	***-2.22***	***-2.09***	***2.80***	1.83
Caudate	0.31	0.05	-1.52	***-2.99***	***-3.28***	1.19	0.06
Putamen	-0.64	-1.32	-0.58	-1.32	-1.53	***8.92***	***10.08***
Thalamus	-0.21	-0.94	***-2.38***	***-2.32***	***-2.95***	1.60	0.80
Hippocampus	***-2.20***	***-2.22***	***-2.95***	***-2.92***	***-2.62***	0.37	-0.76

Brother B	FDG z-scores (cerebellar GM reference)				PiB z-scores (cerebellar GM reference)	
	1996	1998	2006	2009	2009	2012
Frontal	-0.42	-0.58	-0.54	-0.18	-2.94	-2.91
Parietal	-0.22	-0.45	-0.46	-1.84	-0.19	-1.77
Temporal	-0.25	-0.71	-0.23	-0.59	0.53	0.01
Occipital	1.07	0.11	0.41	-0.41	-1.71	-3.83
Anterior cing.	-0.52	-0.79	-0.68	-0.80	1.21	***2.19***
Posterior cing.	-0.46	-0.55	0.26	-0.59	***2.40***	***3.70***
Insula	0.58	-0.68	-0.19	-0.44	1.16	***2.00***
Parahipp.	-0.48	-1.50	-1.22	-1.20	0.56	0.28
Caudate	-0.54	-0.59	0.09	-0.18	-1.17	-1.03
Putamen	-0.19	-0.84	-0.29	-0.71	0.56	1.09
Thalamus	-0.41	-1.01	-0.02	-0.42	***2.82***	***3.54***
Hippocampus	-0.63	-1.83	***-2.24***	***-2.31***	0.68	0.70
